# Progress in Medicine

**Published:** 1900-06-09

**Authors:** 


### Progress in Medicine.

### DISEASES OF THE RESPIRATORY SYSTEM.

**The Therapeutic Value of Heroin.**—Heroin[1] is a diacetic-acid-ester of morphine. It has a marked sedative effect on the respiratory tract, lessens the number of respirations, lengthens inspiration, and increases the amount of inhaled air. The hydrochloride is the most useful preparation on account of its solubility, and, beyond slight dizziness and occasional dryness of the throat, no ill effects have been noticed to follow its administration. From its power of diminishing dyspnœa and alleviating cough, it has proved of great service in asthma and phthisis. Harnack,[2] however, expresses his belief that the drug is a cardiac depressant, and gives rise to muscular twitchings and convulsions. Fulton[3] found heroin in doses of one-sixteenth of a grain every four hours of great service in relieving the incessant cough which sometimes follows influenza, and in securing freedom from cough and rest at night in all stages of phthisis. Its effects were more marked in chronic than in acute bronchitis. In asthma a solution in acetic acid may be injected hypodermically. A *résume* of recent literature bearing upon the therapeutic uses of heroin will be found in recent numbers of "Clinical Excerpts."[4] Carl Mirth recommends the use of this drug in bronchial asthma and whooping cough, in doses of one-twelfth to one-eighth of a grain three or four times daily, or the muriate of heroin in solution may be substituted. Given in these doses, none of the narcotic effects of morphia were observed, nor does it produce constipation or alteration in blood pressure. Heroin has a slight antipyretic action. Its use is advantageous in diseases of the heart and in arteriosclerosis if these affect the respiratory organs in a secondary manner. The above conclusions are borne out by Bongrier,[5] who states that heroin is preferable to codeia inasmuch as it is of stable composition, more powerful in action, less toxic, and in no way diminishes the oxygenation of the blood. Winternitz[6] and Floret[7] also speak highly of the value of this drug, and the latter recommends its use in emphysema. Heroin is best given as a powder with a little sugar; the muriate may be combined with syrup or aqua Laurocerasi.

**Drug Treatment in Phthisis.**—Hawkins[8] gives the following particulars of creosote and its derivatives: Creosote is a mixture of guaiacol, creosol, and other phenols. When decomposed in the intestine the phenols combine with the toxalbumins created by the tubercle bacilli, rendering them innocuous, rapidly oxidisable, and capable of elimination by the kidneys. In this way these symptoms of phthisis produced by the toxins—viz., fever, night sweats, anorexia, and dyspepsia—are relieved, but the destruction of the bacilli must be left to hygienic measures. Creosote also prevents fermentation in the bowels, and by its tonic action improves the general nutrition. It may be given in wine, water, or milk, small doses being first administered until a maximum of 30 grains a day is reached. The various derivatives of creosote, guaiacol, creosote carbonate, guaiacol carbonate, valerianate, phosphate, &c., are chiefly recommended on account of the lessened liability of disturbance to digestion. They are, however, much more expensive than creosote. Dyspeptic symptoms due to the administration of creosote are by no means common. Thus, Bucquoy[9] states that to obtain the full benefit of the drug it must be given in large doses, six to ten grammes (90 to 150 grains) daily, and with these quantities he has found no ill-effects. The odour and taste of creosote and its derivatives are objected to by some patients, and owing to their insolubility in water there is considerable difficulty in dispensing them in an agreeable form. A new preparation, thiocol (potassium ortho-guaiacol sulphonic acid), has neither taste or odour; it is readily soluble, non-toxic, and of great assimilability. Its action is identical with that of the drugs mentioned above. As a powder it may be given in cachet, capsule, or tablet, or it may be used in aqueous solution, combined with orange syrup, or syrup of cinnamon. The dose is 10 grains, gradually increased to 30 grains three times daily after meals. Very favourable reports of the action of this preparation in phthisis have been given by Rossbach,[10] Marcus,[11] and others.[12] Renzi and Boeri[13] recommend a mixture of thiocol and guaiacol named sirolin. Patients treated by this means showed a rapid increase in weight and strength, the quantity of sputum and bacilli decreased, the temperature fell, the urine increased in quantity, and showed increased amounts of sulphates and diminished uric acid. The pulse and respiration were decreased in frequency, and the processes of organic oxidation markedly improved. Each teaspoonful of sirolin contains 5 grs. of thiocol and 3 grs. of guaiacol, and one to five teaspoonfuls were given daily. Wertheimer[14] strongly recommends ichthyol internally in phthisis. Its action closely resembles that of creosote, and it is best given mixed with equal parts of distilled water. One to 10 drops of the mixture may be given three times daily after food.

Dr. William Murrell[5] was one of the first to point out the beneficial effects in phthisis of inhalations of formalin, the 40 per cent. formaldehyde being used, dropped upon lint. These results have been confirmed by Cervello and Lardner Green.[16] Recently Hirschfeld[17] has suggested the use of this drug in the treatment of phthisical sweating. A single limb, or other portion of the body, is painted each night with a solution of 40 per cent. of formaldehyde in an equal quantity of absolute alcohol. When the whole body has been covered, the process is repeated a second or a third time if needful. In this way the sweating is rapidly controlled, and, should it return, is again checked by fresh applications. The unpleasant effect of the fumes may be overcome by holding before the nose and mouth cotton wool moistened with essence of turpentine. Mays[18] gives the following directions for the treatment of hæmoptysis: The patient must be kept at rest in bed, quiet, with an ice-bag to the chest, and to secure repose a quarter-grain of morphine sulphate administered hypodermically, and a 10-grain suppository of asafœtida given at night. The most efficient styptic is strychnine, given in progressive doses of $\frac{1}{32}$—$\frac{1}{10}$ grain hypodermically every six hours. The only nourishment should be cold meat juice.

**Serum Therapy in Tuberculous Disease of the Lungs.**—Although Koch's tuberculin is in great part used for purposes of diagnosis only, reports as to its curative value are still from time to time recorded. Petruschky,[19] after a trial extending over nine years, is of opinion that in 22 cases he has obtained a permanent cure by a graduated plan of treatment, the injections being discontinued with the cessation of the reaction to tuberculin, and being resumed at intervals of three or four months.

Baldwin[20] gives the following list of modified forms of tuberculin: (1) purified tuberculin (T. Pur.), (2) new tuberculin (T. R.), (3) antiphthisin, (4) Hunter's modification, (5) tuberculoplasmin, (6) watery extracts of bacilli without culture medium, (7) tuberculinic acid. Of these the most potent are those which contain dead bacilli, and their action probably depends upon their power of stimulating the secretion of ferments. These ferments attack the toxins formed by the tubercle bacillus and destroy them. In uncomplicated cases of phthisis and lupus good results have followed the administration of tuberculin, but probably this has been due to its action as an irritant, and not to any specific antitoxic property. Mackenzie,[21] in a review of our present state of knowledge with regard to the value and action of antituberculous serums, concludes that none have yet been shown to possess definite curative properties.

[1] Phil. Med. Jour., Oct. 28, 1899. [2] Munch. Med. Wcch, July 4, 1899. [3] New York Med. Jour., Dec. 30, 1899. [4] Clinical Excerpts, July and Nov. 1899. [5] Les Nouv. Remed., Jan., 1900. [6] Therap. Monatsh., Sept., 1899. [7] Les Nouv. Remed., Aug. 8, 1899. [8] The Therapist, May 15, 1899. [9] La Sem. Medic., June 21, 1899. [10] Therap. Monatsh., No. 2, 1899. [11] Würt. Medic. Corres., 1899. [12] Merck's Archives, Oct. 1899. [13] Deut. Med. Woch., No. 32, 1899. [14] Brit. Med. Jour. Epit. 147, June 13, 1899. [15] Brit. Med. Jour., June 28, 1899. [16] Brit. Med. Jour., Jan. 20, 1900. [17] La Semaine Med., Nov. 1, 1899. [18] Merck's Archives, Sept., 1899. [19] Med. Rec., Jan. 27, 1900. [20] Phil. Med. Jour., May 5, 1900. [21] Practitioner, June, 1899.

